# COVID-19 responses restricted abilities and aspirations for mobility and migration: insights from diverse cities in four continents

**DOI:** 10.1057/s41599-023-01721-y

**Published:** 2023-05-18

**Authors:** Dominique Jolivet, Sonja Fransen, William Neil Adger, Anita Fábos, Mumuni Abu, Charlotte Allen, Emily Boyd, Edward R. Carr, Samuel Nii Ardey Codjoe, Maria Franco Gavonel, François Gemenne, Mahmudol Hasan Rocky, Jozefina Lantz, Domingos Maculule, Ricardo Safra de Campos, Tasneem Siddiqui, Caroline Zickgraf

**Affiliations:** 1 School of Business and Economics - UNU-MERIT, Maastricht University, Maastricht, The Netherlands; 2 Faculty of Social and Behavioural Sciences, University of Amsterdam, Amsterdam, The Netherlands; 3 Geography, Faculty of Environment, Science and Economy, University of Exeter, Exeter, UK; 4 Department of International Development, Community, and Environment, Clark University, Worcester, USA; 5 Regional Institute for Population Studies, University of Ghana, Accra, Ghana; 6 Centre for Sustainability Studies, Lund University, Lund, Sweden; 7 School for Business and Society, University of York, York, UK; 8 Department of Geography and the Hugo Observatory, University of Liège, Liège, Belgium; 9 Refugee and Migratory Movements Research Unit, University of Dhaka, Dhaka, Bangladesh; 10 Faculty of Architecture and Physical Planning, University Eduardo Mondlane, Maputo, Mozambique

**Keywords:** Geography, Development studies

## Abstract

Research on the impacts of COVID-19 on mobility has focused primarily on the increased health vulnerabilities of involuntary migrant and displaced populations. But virtually all migration flows have been truncated and altered because of reduced economic and mobility opportunities of migrants. Here we use a well-established framework of migration decision-making, whereby individual decisions combine the aspiration and ability to migrate, to explain how public responses to the COVID-19 pandemic alter migration patterns among urban populations across the world. The principal responses to COVID-19 pandemic that affected migration are: 1) through travel restrictions and border closures, 2) by affecting abilities to move through economic and other means, and 3) by affecting aspirations to move. Using in-depth qualitative data collected in six cities in four continents (Accra, Amsterdam, Brussels, Dhaka, Maputo, and Worcester), we explore how populations with diverse levels of education and occupations were affected in their current and future mobility decisions. We use data from interviews with sample of internal and international migrants and non-migrants during the 2020 COVID-19 pandemic outbreak to identify the mechanisms through which the pandemic affected their mobility decisions. The results show common processes across the different geographical contexts: individuals perceived increased risks associated with further migration, which affected their migration aspirations, and had reduced abilities to migrate, all of which affected their migration decision-making processes. The results also reveal stark differences in perceived and experienced migration decision-making across precarious migrant groups compared to high-skilled and formally employed international migrants in all settings. This precarity of place is particularly evident in low-income marginalised populations.

## Introduction

The SARS-CoV-2 pandemic has greatly affected patterns of human mobility in every corner of the world. The introduction of travel restrictions and border closures, alongside reduced economic opportunities, caused a substantial downturn in international migration, as evident in published figures from mid-2020 onwards (UN DESA, 2021). Similarly, emerging findings suggest that internal migration was also disrupted by stringent population movement controls, businesses shutdowns and social distancing; all of which combined altered individual decisions associated with life course transitions (see González-Leonardo et al., 2022; Stawarz et al., 2022). High levels of involuntary displacement and rising global mobility have increased the risks of pandemics, from H1N1 to Ebola, because they make the world more interdependent and connected, as part of fragility in global health systems (Greenaway and Gushulak, 2017). Marginalised populations that include involuntary migrants have been shown to have greater exposure and higher mortality and negative outcomes from COVID-19 in many countries (Greenaway et al., 2020).

Building on these important insights, more systematic and comparative research is needed to explore the mechanisms through which the pandemic affected migration decisions at individual levels. Given the observed macro-level trends and outcomes, this paper seeks to identify generalized mechanisms through which the global pandemic affected individual migration decisions, made by previous and potential migrants, which resulted in these altered global migration flows. In this article, we use an aspiration-ability framework (Carling, 2002, 2014; Schewel, 2020) to understand how individual mobility decisions were affected by the pandemic and to identify the mechanisms that affected the mobility decisions of self-identified international and internal migrants, as well as those of non-migrant individuals, in global urban contexts.

Many studies have focused on migrants’ increased vulnerabilities during the pandemic, most often zooming in on specific migration groups (e.g. labour migrants, students, rural–urban migrants) (Elisabeth et al., 2020; Nimer and Rottmann, 2021; Schotte et al., 2021), and with an emphasis on the most socially vulnerable (e.g. irregular migrants, or displaced and refugee populations) (Greenaway et al., 2020; San Lau et al., 2020; Raju et al., 2021; Suhardiman et al., 2021). In some cases, the emerging evidence on migrant vulnerability has shown how the COVID-19 pandemic has had significant impacts on migrants’ livelihoods, which, in turn, affected migration aspirations and migration decisions. In Singapore and Thailand, for example, Suhardiman et al. (2021) observed how the pandemic altered migration aspirations by affecting migrants’ livelihoods. These impacts on migration aspirations differed according to migration status (regular or irregular), access to formal work, and level of social protection. Yet, focusing on vulnerable migrant populations does not allow for a distinction between the vulnerabilities specific to marginalised populations in particular areas and the vulnerabilities shared by migrants or non-migrants more generally.

The objective of this study is to identify and explore the different mechanisms through which the pandemic affected individual mobility decision-making practices. To do so, we draw inspiration from the aspiration-ability framework that perceives migration or mobility decisions “as a function of aspirations and capabilities to migrate within given sets of perceived geographical opportunity structures” (de Haas, 2021, p. 2). Following this framework, a migration or mobility decision is, firstly, dependent on the “immigration interface”; the macro-level context which determines the “barriers and requirements” for migration (Carling and Schewel 2018, 947). Secondly, migration decisions are a two-step process, comprising, firstly, the aspiration to migrate and, secondly, the ability to realize this move (Carling and Schewel, 2018). The aspiration-ability framework, therefore, allows us to identify three mechanisms through which the pandemic affected migration decisions: (1) through the direct impacts of barriers to movement (e.g. travel restrictions and border closures), (2) through the impact of individual economic circumstances on mobility decisions (i.e. abilities to move), and (3) through the impacts of the pandemic on aspirations to move.

By focusing on both structure (context) and agency (aspirations and abilities), we look beyond individual circumstantial factors related to the COVID-19 crisis and pay attention to structural factors that reduce people’s abilities and increase migrants’ precarity of place (Banki, 2013), understood here as migrants’ specific vulnerabilities that lower their choice and agency to stay in their main place of residence. We suggest that the generalized migration-oriented responses to the COVID-19 pandemic we identify are dependent on structural factors as well as the manifest abilities of individuals and their agency—people’s abilities, representing the freedom of choice on what they manage to do or to be given what they have, and their personal and social circumstances (Sen, 1999). In terms of agency, we consider that migrants may have varying levels of agency in their migration decisions, following Hugo’s (1996) definition of population mobility as “a continuum ranging from totally voluntary migration, in which the choice and will of the migrants is the overwhelmingly decisive element encouraging people to move, to totally forced migration, where the migrants are faced with death if they remain in their present place of residence” (Hugo, 1996, p. 107). To capture the wide range of mobility options that individuals have, we look at aspirations and decisions to move or to stay put in the short and long term, and we also consider temporary moves (e.g. temporary return, circular mobility) by people with attachments in multiple places within one country or transnationally.

For our analyses, we use new and unique comparative evidence of the experiences of migrants and non-migrants in six cities across four continents—Accra, Amsterdam, Brussels, Dhaka, Maputo, and Worcester. These cities represent small and large cities across the Global North and Global South, with varying population sizes, and varying trajectories of dominant migration. The data were collected through in-depth interviews with 47 migrant and non-migrant residents during the SARS-CoV-2 outbreak in 2020. The data is designed for an analysis of how the pandemic affected individual mobility decisions, through the three identified mechanisms described above.

During the COVID-19 pandemic in 2020, when the data were collected, all of the cities were under significant travel restrictions but had very different policies in supporting lost incomes for those whose livelihoods were directly affected by public health interventions. These policies ranged from furlough schemes and direct wage support in Amsterdam and Brussels to little or no income support in Maputo and Dhaka. The multi-sited character of this study thus provides a diversity of perceptions and experiences of migration during the pandemic that reflects the impacts of the COVID-19 crisis in cities with diverse migration histories and profiles.

The next two sections present an overview of the evidence to date on the impacts of the COVID-19 crisis on migrant vulnerability and mobility decisions, the theoretical approach and research questions. The third section presents the data and methods and the developments around COVID-19 in the six cities at the time of the study. The results document the three aspects of consequences for migrants that increased their precarity of place. The discussion highlights the long-term and wider implications for social differentiation and recovery.

## How migration has been affected by COVID-19

The COVID-19 pandemic has thrown into sharp relief how human short- and long-distance mobility initially enabled the spread of the virus globally. Migration flows were significantly altered as a result of the pandemic. A diversity of policy responses to the threat of the spread of the virus, including lockdowns and bans on travel within and between countries, had direct impacts on the intensities and directions of mobility patterns and internal and international migration flows. The United Nations Department of Economic and Social Affairs revealed that the growth in the stock of international migrants may have been reduced by around two million (or a 27 per cent decline from the growth expected since mid-2019) by mid-2020 as a consequence of the pandemic (UN DESA, 2020). Some migration commentators are going further to suggest that the pandemic may represent an inflection point: that international movement at the global scale may have peaked before the pandemic (Gamlen, 2020).

In addition, perceived risk of virus transmission, at least in early stages of the pandemic in 2020, led to stigma and blame on migrant populations (San Lau et al., 2020). The fear of the virus spreading, of international or local disease transmission, further marginalized migrant populations. The biosecurity framing of public health and disease control has been argued to create unforeseen and unpredictable social outcomes (Lentzos and Rose, 2009). As such, the COVID-19 crisis amplifies many elements of social inequality for migrant populations in cities, thus bringing to light long-standing issues of policy and social protection associated with migration, particularly for low-income migrants in informal settlements, and active in informal and casual work (Raju et al., 2021; Rao et al., 2020; Siddiqui et al., 2021). Many migrants experience spatial and social marginalisation in the places they move to, which manifests as low life satisfaction, higher levels of stress, and perceived insecurity in low-income settings (Adger et al., 2021; Siddiqui et al., 2021). Socially excluded migrant populations may experience negative mental health outcomes which may be exacerbated by limited labour rights, social stigma and inequality (Li and Rose, 2017; Richaud and Amin, 2019). Migrants can end up jobless and with limited or no access to formal social protection in their place of destination (Sabates-Wheeler and Feldman, 2011) and limited opportunity for return migration to their place of origin (Içduygu, 2020). All these factors are likely to contribute to migrants’ precarity of place, which is not necessarily related to labour precarity alone (Banki, 2013).

The COVID-19 crisis has had significant impacts on migrants’ livelihoods at all income levels, which, in turn, often reconfigured migration aspirations and migration decisions (Suhardiman et al., 2021). For low-income marginalised populations, economic crises and downturns generally result in significant risks of falling into poverty through unemployment (Aiyemo, 2020). Their vulnerability can increase depending on factors such as gender, age, ethnicity or when social networks in the place of residence are limited (IOM, 2019). In line with this work, there is now growing evidence that the economic lives of migrant populations were disproportionally affected by the direct and indirect effects of the pandemic. For instance, some migrants were more likely to contract the virus due to their living and working conditions, they had less access to health care and in several countries, the COVID-19 crisis particularly affected industries where migrants are highly represented, such as the health, social care, hospitality and food industry sectors (Fernández-Reino and McNeil, 2020; Guadagno, 2020).

Economic downturns trigger shifts in migration processes with short and long-term impacts on source and destination economies. The Asian economic crisis of the late 2000s, for example, led to significant urban-to-rural return migration, reversing decades of prior movements and truncating land use transformation processes (Gödecke and Waibel, 2011; Rigg et al., 2018). The unprecedented level of travel restrictions implemented in many countries due to COVID-19 affected migrants in multiple ways. For example, border restrictions and closures trapped low-skilled migrant workers who often faced increased economic hardship (IOM, 2021). Furthermore, without access to social welfare, migrants in the Gulf and parts of South-East Asia were often excluded from access to public health and unemployment benefits, resulting in increased vulnerability (ADBI, 2021; IOM, 2021). There is a growing body of evidence on the short-term effects of the pandemic on forced immobility, for instance in China (Li et al. 2021). In Singapore and Thailand, Suhardiman et al. (2021) observed that the COVID-19 pandemic affected migrants’ livelihoods and subsequent migration aspirations differently according to migration status (regular or irregular), access to formal work and level of social protection. The longer-term effects of COVID-19 on migration processes are linked with hardening of international borders through artificial intelligence as well as socioeconomic consequences associated with changes in labour markets and remittance corridors (IOM, 2021).

Furthermore, as part of the migration decision-making process, ambivalences might play a role in the constant redefinition and re-routing of migration individual projects (Boyer, 2005; Jolivet, 2020; Schapendonk et al., 2020). For example, significant technological advances might enable prospective migrants to access work and education opportunities through digital platforms (IOM, 2021). Migrants and non-migrants may experience ambivalent aspirations to stay put or to migrate and preferences around mobility may change over time as the effects of the COVID-19 crisis unfold, leaving people to balance economic factors, formal and informal social protection resources, and their quality of life.

## Restrictions on movement, abilities and aspirations to move

**Fig. 1:**
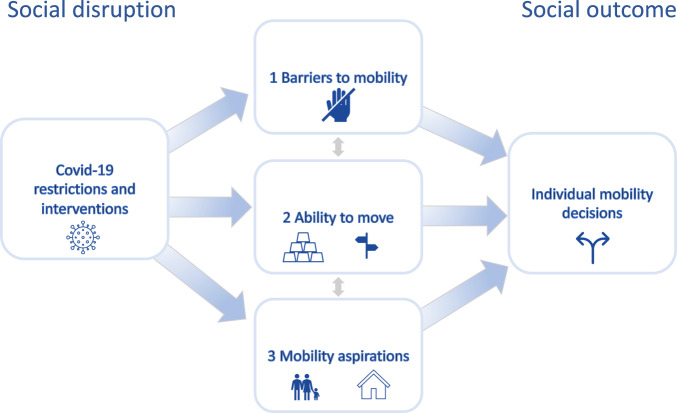
Barriers to movement, capacity to move, and changing aspirations affected mobility outcomes. Individual decisions on current and future movement (right hand side) are affected by COVID-19 policies and restrictions (left hand side) through the three mechanisms of barriers to mobility, resources and ability to move, and altered aspirations.

To study the impact of the COVID-19 pandemic on experiences of mobility we analyse three mechanisms through which we suggest the pandemic could have affected individual decision-making practices (Fig. 1). First, the barriers refer to the macro-set of opportunities or the “given sets of perceived geographical opportunity structures” (de Haas 2021, p. 2), in which mobility decisions are made. Second, abilities to move are based on the concept of abilities, which are the effective opportunities available to individuals to pursue valued functioning, or states of ‘being’ and ‘doing’ (Robeyns, 2006; Sen, 1985). The evidence on the interplay between abilities and mobility or migration has mainly focused on the lack of ability or capability to move that leads to involuntary immobility (Carling, 2002; Collyer et al., 2012; Lubkemann, 2008) or on how increased abilities and life aspirations lead to increased aspirations to migrate (de Haas, 2003, 2006, 2014; Suhardiman et al., 2021). A further dimension is the effect of migration on the capacity to aspire (Czaika and Vothknecht, 2014) or on the contrary, how decreased abilities brought about by the COVID-19 pandemic reduce capacities to aspire (Suhardiman et al., 2021). We frame this research by observing that the COVID-19 pandemic and its associated governmental responses impose various constraints on individuals’ abilities to choose to move or stay.

Third, mobility aspirations are referred to as the belief that migration is preferable to staying (Carling, 2002; Czaika and Vothknecht, 2014; de Haas, 2014; Carling and Schewel, 2018). Mobility aspirations may, firstly, be affected by the pandemic through increased perceptions of risk associated with mobility. Clearly, the pandemic has raised individuals’ fear of health deterioration through the risk of infection. But government responses to the pandemic, such as restricting movement, have not only affected people’s abilities to move by reducing their choice set (through individuals’ perceptions of risk) but also involuntarily through regulations and restrictions. As such, barriers to mobility as well as abilities to move have consequences for aspirations to move. At the individual level, changes in abilities to move to affect mobility aspirations, which in turn have a knock-on effect on how migration outcomes are perceived among peers through mechanisms of social diffusion (Carling and Collins, 2018).

## Data and methods

The research design was to elicit data on the range of experiences among a diverse set of migrant and non-migrant populations in diverse urban settings. Primary data was collected using semi-structured interviews conducted with participants in Accra, Amsterdam, Brussels, Dhaka, Maputo, and Worcester (US). The six cities were selected to ensure maximum variation in terms of areas of origin and destination of migration.

### Site selection

Known figures on COVID-19 infections in the place of residence and policy responses to the COVID-19 pandemic during fieldwork could have influenced participants’ perceptions and experiences of migration in 2020. Table 1 contains country-level data related to the city sites and includes information reported to and published by WHO (2021). In Mozambique, the level of identified COVID-19 infections was relatively low; Belgium and the Netherlands were out of their first wave; in Ghana, infections were increasing; and while Bangladesh was at the peak of its first wave, the United States was getting out of a second wave—in the state of Massachusetts where Worcester is situated, detected COVID-19 infections were relatively low compared to country-level figures (The New York Times, 2021). When the fieldwork started, only Mozambique had more stringent biosecurity measures than in March 2020. In all sites, schools were closed at least for some levels of education. There were also closings in some sectors of occupation, the organisation of public events was not permitted and there were restrictions on the maximum number of people allowed in social gatherings. Restrictions on public transport use were recommended in Bangladesh and the United States. In the Netherlands, Mozambique, and the United States, staying at home was recommended, while in Bangladesh this was a requirement with some exceptions. In terms of social protection, policies for supporting income were in place in Belgium, the Netherlands, and, to a lesser extent, the United States, but not in the other three countries (Hale et al., 2020).

**Table 1:** Characteristics of six sampled cities and national responses to the COVID-19 pandemic at the time of data collection mid-2020.

City	Population (m)	Migration trajectory	Restrictions on public transport (mid-2020)	International travel measures (mid-2020)	Income support
Dhaka	20.3	Main destination of all types of migrants. Growth of slums where 53% of residents had migrated from the rural hinterlands and smaller urban districts	Recommended closed	Complete travel ban	No formal support
Brussels	1.2	More than half of its population is foreign-born. Aside from migrations linked to labour migration (Southern Europe, North Africa, Turkey) or its colonial past (like DRC), there is a large representation of EU-born citizens working for the EU institutions	No measures	Complete travel ban	>50% lost income
Accra	2.6	Primary destination of the majority of Ghana’s internal migrants and a major destination for international migrants from the West Africa sub-region. New migrant populations are clustered in high-density informal settlements	No measures	Border closures	No formal support
Maputo	1.7	Rapid growth of the city as a result of migration flows from rural areas associated with political instability, war, poverty or unsuccessful agricultural policies. Migration is linked to factors including job security, aid, frontline services, and economic opportunities in both informal and formal sectors.	No measures	Border closure	No formal support
Amsterdam	0.9	Major destination for domestic and international migrants with established diasporas from Turkey, Morocco, Suriname, the Antilles, and Sub-Saharan African countries.	No measures	Complete travel ban	>50% lost income
Worcester	0.2	A historic migration destination as a manufacturing hub giving rise to a diverse ethnic and racial population. Current domestic and international migration is linked to biotech, education, and employment opportunities, as well as small numbers of refugees.	Recommended closed	Complete travel ban	>50% lost income

Source: Various including COVID-19 Government Response Tracker (Hale et al., 2020).

### Interview participants

The study reported here originally targeted a sample to explore perceptions and meanings of social, environmental and community elements of sustainability for newly migrated urban populations. Hence the participants were recruited from July 2019 onwards using maximum variation sampling, with the aim to ensure diversity in socio-economic characteristics, experiences in urban environments, and perspectives on sustainability. The first wave interviewed participants face-to-face. In the present study, we use data collected subsequently collected from a subset of 47 participants who agreed to participate. We re-interviewed these participants in 2020 during the COVID-19 pandemic. The participants were purposively selected based on their migration experience and were from a diverse range of regions of origin based on place of birth, and a parallel smaller cohort of non-migrants (see Table 2). We distinguished between non-migrants, internal migrants, and international migrants. Non-migrants were men and women born and raised in the city of residence. This category could include people who were born and raised in the city, migrated (internally or internationally), and returned to the city. Internal migrants were those who moved from other parts of the country (rural or urban) to the city. Internal migrants were excluded in Amsterdam and Brussels because rural-urban differences are smaller in Belgium and the Netherlands than in the other research sites. International migrants were people who migrated to the city from another country. Internal and international migrants had to reside for a period ranging from one to five years in the cities under study before data collection. The 12-month period aligns with internationally applied definitions of long-term migration (UN, 1998).

**Table 2:** Characteristics of 47 participants with varying socio-demographic profiles.

Migration profile	City of residence	Year of birth	Gender	Country of birth	Years of education	Main occupation	Reference
High-income internal migrants	Accra	1990	Male	Ghana	9	Electrician and lotto agent	ACC-M1-M-AA-03
	Accra	1989	Female	Ghana	15	Caterer	ACC-M1-F-AA-04
	Accra	1995	Female	Ghana	16	Call centre agent	ACC-M1-F-MA-06
	Dhaka	1983	Female	Bangladesh	15	Administrative staff	DHA-M1-F-MH-04
	Dhaka	1990	Male	Bangladesh	20	Research officer	DHA-M1-M-SM-09
	Dhaka	1992	Male	Bangladesh	20	Research officer	DHA-M1-M-GM-10
	Maputo	unknown	Male	Mozambique	18	Architect, self-employed	MAP-M1-M-DM-08
	Worcester	1961	Female	USA	19	CEO in mental health organisation	WOR-M1-F-JL-07
Low-income internal migrants	Accra	1986	Male	Ghana	9	Fashion designer	ACC-M1-M-AA-02
	Accra	1992	Female	Ghana	14	Unemployed	ACC-M1-F-MA-03
	Dhaka	1959	Male	Bangladesh	0	Cleaner in hotel	DHA-M1-M-MH-02
	Dhaka	1990	Female	Bangladesh	3	Unknown	DHA-M1-F-MH-03
	Maputo	1994	Male	Mozambique	17	Not employed, nor looking for a job	MAP-M1-M-SS-04
	Maputo	1996	Male	Mozambique	18	Not employed, nor looking for a job	MAP-M1-M-DM-09
	Worcester	1974	Female	USA	17	Hairstylist	WOR-M1-F-JL-05
	Worcester	1999	Female	USA	15	Student job	WOR-M1-F-JL-09
High-income international migrants	Accra	1989	Male	Nigeria	14	Businessman selling phones and accessories	ACC-M2-M-AA-01
	Amsterdam	1981	Female	Bangladesh	19	PhD candidate	AMS-M2-F-RT-03
	Brussels	1989	Male	India	15	PhD candidate	BRU-M2-M-SND-N5
	Brussels	1991	Male	U.S.A.	15	Political consultant	BRU-M2-M-SND-N2
Low-income international migrants	Accra	1990	Male	Togo	14	Bouncer in casino	ACC-M2-M-MA-04
	Worcester	1973	Female	Iraq	17	Case manager	WOR-M2-F-JL-10
	Amsterdam	1978	Female	Colombia	19	Cook	AMS-M2-F-MV-01
	Worcester	1981	Male	Cameroon	17	Graduate student	WOR-M2-M-JL-11
	Dhaka	1983	Male	Nepal	20	Not employed, nor looking for a job	DHA-M2-M-URD-11
	Maputo	1982	Female	Rwanda	15	Self-employed	MAP-M2-F-SS-10
	Maputo	1990	Female	DRC Congo	3	Trader/dealer	MAP-M2-F-AG-13
	Accra	1986	Female	Cote d’Ivoire	14	Trading in provisional store	ACC-M2-F-AA-05
	Accra	1991	Female	Nigeria	18	Unemployed	ACC-M2-F-MA-07
	Dhaka	1945	Male	UK	15	Unknown	DHA-M2-M-MH-05
High-income non-migrants	Worcester	1954	Male	USA	15	Carpenter, self-employed	WOR-NM-M-JL-08
	Accra	1981	Male	Ghana	16	District disaster officer	ACC-NM-M-MA-05
	Amsterdam	1969	Male	Netherlands	17	Entrepreneur	AMS-NM-M-KB-01
	Amsterdam	1964	Female	Netherlands	18	Freelance	AMS-NM-F-RT-04
	Dhaka	1985	Female	Bangladesh	20	Higher Assistant, Dhaka Education Board	DHA-NM-F-YA-04
	Worcester	1952	Female	USA	22	Violine teacher	WOR-NM-F-JL-04
	Worcester	1935	Male	USA	19	Retired	WOR-NM-M-JL-01
Low-income non-migrants	Accra	1971	Male	Ghana	9	Lotto agent	ACC-NM-M-AA-08
	Worcester	1991	Female	Ukraine	15	Not employed, nor looking for a job	WOR-NM-F-JL-02
	Brussels	1999	Male	Belgium	15	Student job (bar)	BRU-NM-M-SND-N3
	Accra	1989	Female	Ghana	15	Unemployed	ACC-NM-F-AA-07
	Accra	1975	Male	Ghana	9	Unemployed	ACC-NM-M-AA-06
	Brussels	1994	Male	Belgium	15	Unemployed	BRU-NM-M-SND-N9
	Maputo	1998	Female	Mozambique	12	Unemployed	MAP-NM-F-RD-02
	Maputo	1993	Female	Mozambique	12	Unemployed	MAP-NM-F-RD-05
	Accra	1988	Male	Ghana	18	Unknown	ACC-NM-M-MA-01
	Dhaka	1991	Female	Bangladesh	8	Worker in quality section (Garments)	DHA-NM-F-J-07

Participants were aged between 18 and 85 years and in the migrant sub-sample all had migrated to the city of residence as adults. Participants were recruited in 2019 through personal contacts, community group leaders and snowball sampling. The interview guide included questions for the migrant sample to reconstruct participants’ migration history (including decisions to remain in place) that could provide a better understanding of participant’s frames of reference, geographic comparisons and changes in perceptions, attitudes and behaviours over the migration process. The data collected between June and September 2020 with 47 participants focussed on the impact of the COVID-19 pandemic on the participants, which included a section on their migration aspirations and decisions and socio-demographic characteristics.

### Interview procedures and data analysis

Interviews were conducted remotely from May to July 2020 during the pandemic using video calling or mobile phones. Phone interviews complied with strict ethical guidelines for conducting research during the Covid-19 pandemic. The interviews were recorded, transcribed and translated into English and analysed using a so-called hybrid process of inductive and deductive theme coding (Fereday and Muir-Cochrane, 2006). We designed a codebook to code the interviews using the software QDA Miner. This software has a free version (QDA Miner Lite) that made the resulting coding easily accessible to a team of researchers affiliated with different institutions with access to diverse software. We started with deductive coding, based on the codebook in order to organise the data. We completed the deductive coding of overarching themes with a second phase of inductive coding.

### Sample description

As described, the sample contains 47 individuals with varying socio-demographic profiles (Table 2). First, the internal migrant respondents are evenly split among higher and lower income levels, while those with higher income profiles did not necessarily have more years of education. The two internal migrant respondents in Maputo, for example, achieved university-level education but were both unemployed, while two highly educated internal migrants in Worcester were employed as relatively low-status and low-income hairstylists and student workers, respectively. Internal migrants with fewer years of education included a male hotel cleaner, a male fashion designer, and an unemployed woman. Among the higher income category, those with advanced degrees comprised two male research officers in Dhaka, an architect in Maputo, and the head of a non-profit organisation in Worcester.

The sample had fewer international migrants of high income: an American political consultant and an Indian doctoral candidate in Brussels, both men; a Nigerian businessman in Accra, and a Bangladeshi doctoral student in Amsterdam. Two doctoral student respondents were employed and comparatively well-paid by their universities. The low-income international migrants were nearly all well-educated with the exception of a female Congolese trader in Maputo. A majority of the low-income international migrants were women, with a variety of occupations ranging from social service providers, cook, bouncer, and trader, to graduate student, self-employed, and unemployed. The Accra and Maputo respondents were all from other African countries, while Worcester and Amsterdam’s respondents included a refugee from Iraq, a Colombian migrant, and a Cameroonian student. Dhaka’s two international migrant respondents were from Nepal and the UK.

The number of non-migrant respondents in the higher-income category was slightly smaller than the number in the lower-income category. Of the lower-income non-migrants, the majority were unemployed, while the rest included a Belgian non-migrant working in a bar, a Ghanaian lotto agent, and a Bangladeshi garment worker. In the higher occupation category, non-migrants had a range of jobs including a self-employed carpenter and a violin teacher in Worcester, an entrepreneur and a freelancer, both in Amsterdam, an administrative assistant in Dhaka, and a District Officer in Accra.

## Results: Mobility decision-making during Covid-19

Barriers to movement, abilities to move, and aspirations for mobility are differentiated across social status and class. Migrant and non-migrant respondents representing various occupational and educational levels across the six different locations experienced differential impacts in terms of the barriers they faced, and their abilities and aspirations regarding mobility decisions in the time of Covid. Here we first describe common mechanisms through which COVID-19 affected individual decision-making across the six diverse localities around mobility trajectories. We then use context-specific demographic information on the type of occupation, employment context, and years of education, with respondents divided into relatively higher- or lower-income categories.

### Movement barriers affecting decision-making

The data show how the barriers related to the COVID-19 crisis disrupted longer-distance migration and produced discontinuities in migrants’ multi-sited arrangements. This reduced individual abilities to stay put or migrate, but also to take any migration-related decisions. Emergent consequences in migration aspirations and decisions range from immobility to reconsiderations of long-term migration decisions.

Respondents identified three types of barriers to their decisions to move caused by the COVID-19 pandemic: (a) direct restrictions due to lockdown policies limiting mobility; (b) fear of contracting the SARS-CoV-2 virus; and (c) obligations to stay put in order to protect family members with poorer health conditions or to comply with expectations of other household members. Barriers affecting decision-making for high-income international migrants varied by location and type of occupation. An Indian doctoral student in the high-income group described how he was affected by local restrictions in Brussels:

“I think there was a period during the serious lockdown when I felt kind of closer with people because I think people were more willing to have full conversations, or go on walks or do sort of, I would say, low-key activities or keep communications because everyone was a bit more lonely. And now, I think, their opening up, I think… I would say I feel a bit more isolated because everyone is going about their lives a bit more like normal now. And my life still feels very much on hold”. (BRU-M2-M-SND-N2*).

Lockdown policies affecting mobility had further consequences for income generation. For example, a high-income Nigerian businessman in Accra found his ability to move to conduct his import and export business stalled: When asked where he would want to move to, he replied, “I do business in China. I import my goods from China and send them to Cameroun and Nigeria but I can’t do it anymore”. (ACC-M2-M-AA-01) Tougher and constantly changing travel and entry policies in many countries to manage the threat of COVID-19 point indeed to new barriers and obstacles that affected migrants with trans-national lifestyles. This was the case for a high-income migrant born in Bangladesh living in Amsterdam, who hesitated between returning temporarily to Dhaka to emotionally support her mother during the COVID-19 crisis and staying in Europe to avoid the risk of losing her residence permit that she struggled to obtain:

“Until I complete my PhD out here, I might not be able to move out of the Netherlands. Because if I leave, I may not be able to come back. Because Bangladesh is so far behind in, like, dealing with the COVID-19. (…) So, zone wise, if that country is never under control with its transmission, other countries are not going to open commercial flights. Why would northern Europe, which has flattened the curve, why would they open themselves up to countries which haven’t, (…) I really don’t know when the next time I’ll be able to go back home, you know? So, the thing is that I literally have to finish and then make this whole decision that: do we stay on or do we actually go? And if we leave, then we leave for good”. (Amsterdam international migrant AM2).

Lower-income international migrants faced barriers to travel to their countries of origin due to travel bans rather than a lack of resources. A graduate student in Worcester was unable to return to Cameroon for the funeral of his mother:

“[I’m] here and my family back in Cameroon. So, let me start by myself here and then I will talk about what is happening back in Cameroon. So right now, here I actually had to travel, especially when I lost my mother. But I was not able to do that because of the closures of the borders and the restrictions on the flights. Yeah, I think I am just getting used to it. That was something I wanted to achieve but could not achieve because of the lockdown. Yeah”. (WOR-M2-M-JL-11).

Similarly, a low-income international migrant woman from Cote d’Ivoire in Accra described her inability to travel: “Right now how the sickness has taken over our lives, my father is dead and I can’t go. … I am praying and waiting for the borders to be opened. It is hard”. (ACC-M2-F-AA-05).

Everyday multi-sited arrangements of migrants and their family members became disrupted at all income levels. For instance, internal migrants were unable to travel from Dhaka to their regions of origin to celebrate the religious Eid holidays with family members, both a low-income cleaner (DHA-M1-M-MH-02) and a high-income administrator (DHA-M1-F-MH-04) explained. Transnational health care arrangements were also hampered by the crisis; one lower-income internal migrant in Accra whose son received regular herbal treatments in Togo, where the brother of the respondent lived, was unable to travel since the pandemic outbreak (ACC-M1-M-AA-03).

Perceptions of increased risks and uncertainties of migration are often linked to a mix of biosecurity, infrastructure, and economic factors. A low-income internal migrant in Maputo explained how his decision-making was affected by his family’s fear of the virus: “The people in my house were afraid that I was going to travel. In some ways it was a restriction” (MAP-M1-M-SS-04). Another low-income migrant in Dhaka described how he decided to curtail his mobility:

“In fact, I did not intend to go anywhere since the corona virus outbreak. Once I intended to go to my village. As the situation got worse and caused restrictions on transportation, I could not move. (…). It seems too that if I stay in Dhaka, I could be safe while I am here. If I would move to my village, it would be very difficult for me to adjust to new changes. That is why I did not go to the village”. (DHA-M1-M-GM-10).

High-income non-migrants across the six sites recognized their privilege and choice in the matter of having the economic security to stay where they were already living. Many described their lives under the lockdown as relatively unchanged, although biosecurity fears and isolation for some tempered the reported benefits of reconnecting with friends, slowing down the pace of life, and enjoying access to outdoor spaces. A high-income non-migrant woman living in Worcester described the effect of the Covid lock-down on her and her family’s work, housing, health and living conditions:

“Well, I think that in a way we might be healthier than we were before this because we can we have more time to exercise. We’re not going out and eating junk food. We’re buying stuff and cooking it at home. The work, as I said, I’m teaching. I’m continuing my teaching on Zoom. So that has not changed for the financial aspect. It’s changed my style of teaching, but it’s not changed. Nothing’s really changed in this in that sense’.(WOR-NM-F-JL-04*).

A non-migrant woman in Amsterdam suggested that while her life may have changed, her life satisfaction had not. “I did make choices that were different. But no, it didn’t have any influence on my life satisfaction. I rather thought, wow this is an enormous wake-up call, to go back to… well that’s my thing of course, I do a lot with spirituality, so it was a nice chance to unwind” (AMS-NM-F-RT-04-RT).

For non-migrants in lower-income categories, the experience of life satisfaction during the pandemic restrictions was more mixed. Non-migrants living in countries with better social safety nets experienced isolation and fear, but not desperation as some of the low-income residents in Maputo and Accra explained. For example, a woman in Accra shared her experience of the effect of Covid:

“I am out of job. Things were ok before the covid came. I was doing something small but due to the covid I have stopped so it has had effect. [Before,] I was setting questions for a particular school which was given me some income but because of the covid, the schools have been closed. I now rely on my mum to survive”. (ACC-NM-F-AA-07*).

A young non-migrant man living in Brussels was thankful that he was not at as high a risk as others, despite his dissatisfaction with the Belgian response to Covid. When asked whether he thought living in Belgium had been good for him, or another place was better, he shared that,

“On an individual level, I think it’s the same for me to live in Belgium, than in friends or Germany during the crisis. But obviously I just said that, like, the global strategy wasn’t as good in Belgium, than in other countries. But its impact on the oldest people and like the vulnerable people– that doesn’t impact me because I’m young. And even if I’ve been infected with the coronavirus. It’s not, it’s not, it’s not lethal for me. I’m not into, not in a risk group, you know, like the risk group is the oldest people and the vulnerable and I’m not in it. So for me, it’s the same. If I was like, 78 years years old, it would have been different”.

The COVID-19 pandemic has revealed underexplored dimensions of what Carling (2002) categorised as physical dangers perceived to constrain migration. Such constraints are linked to the biosecurity dimension of the COVID-19 crisis rather than to the dangers of exploitation, trafficking, or irregular migration.

### Curtailed abilities to move

The most obvious effect of the restrictive mobility policies on our respondents was immobility. The lack of ability to move led to involuntary immobility—based on observations, this was perhaps less the case in Dhaka. However, our results indicate differences in how higher and lower-income migrants, both international and internal, experienced the curtailed ability to move. For example, higher-income international migrants, regardless of setting, did not refer to financial hardship that curtailed their movement; rather, it was a lack of ability to make choices about their next steps. A Bangladeshi doctoral student in Amsterdam described her decision-making process thus:

“I mean, there are times when I did very seriously think of just packing, seriously, think of just packing up everything and going back because my mom is by herself, so. I mean, she has help and things like that, but I just hate the fact that she was completely on her own. But everybody was like, look, don’t do that because the chances are that… I mean, A: the whole process is so difficult. And, you know, especially with the PhD right now. I’m at that place where I’m about 30 to 40 percent done and for me to sort of, like, pack up everything and then go back, it’s just. Yeah, so that. But other than that, no, but we’ve been in terms of within Amsterdam, we’ve been OK. We are where we are, where we are”. (AMS-M2-F-RT-03-RT).

For lower-income international migrants, the lockdown restrictions also brought about involuntary immobility, which caused high levels of stress. None of these international migrants, however, discussed changes to their economic ability as getting in the way of achieving their goals. A Cameroonian graduate student in Worcester said:

“Yeah, so I wanted to move, to travel to my country. I was not able to do that. That intention to move was even before the pandemic situation because after my spring semester, after every semester, my goal was to visit my family and spend some time and come back. Unfortunately, this time I was not able to do that because of the lockdown”. (WOR-M2-M-JL-11).

Perceptions of immobilization were not limited to high-income migrants. For example, a lower-income internal migrant in Maputo said that he felt trapped by the lack of freedom of mobility: “Human beings, by their very nature, are not made to feel trapped” (MAP-M1-M-DM-09). But the livelihoods of lower-income internal migrants were more clearly a factor in their inability to move. Other lower-income internal migrants were particularly affected by the lack of ability to move when they or their family members were stranded away from their main place of residence, with significant negative consequences to their livelihoods. When asked about how he was managing his day-to-day expenditures and food costs, a lower-income Ghanaian electrician responded:

“I became a bit free when the lockdown was eased. As I told you, it came impromptu, so I didn’t prepare, but now am able to go out and look for money. Some of my siblings came from Koforidua before the lockdown and they couldn’t go back, so I had to feed them”. (ACC-M1-M-AA-02).

The travel restrictions brought on by the pandemic changed the ability of high-income non-migrants to travel for work and for holiday but also altered their perspectives about their mobile livelihoods and lifestyles. A non-migrant anthropologist in Amsterdam shared with us that she thought to herself, “what a silliness, last year I was in Namibia, why do I have to be in Namibia? Why do we all have to go all the time a weekend to Rome, and this and that. So I’m starting to think differently about traveling. Why do we have to do that?” (AMS-NM-F-RT-04-RT) Meanwhile, another non-migrant from Amsterdam had to rethink his family holiday plans due to the pandemic but was able to justify it in terms of his business needs:

I did want to go on a holiday in April. So we thought, we’ll leave the 25th of March and stay in Thailand for a month, see you later. We’ll follow the news from there, we thought. But that was canceled, we couldn’t go there. And everything was closed so then it’s no fun either actually. But in the first instance we did have the plan to get away for a month. If we have to close anyway, let’s go on holiday now then. Then we don’t have to hire someone for the holiday months, and you move the costs a bit. (AMS-NM-M-KB-01-KB).

Low-income non-migrants experienced the pandemic’s effect on their ability to move differently than the high-income non-migrants since their resources to enable them to move were negatively affected; additionally, these respondents expressed safety concerns about moving to other countries due to the pandemic. When asked whether they had to or wanted to move since the outbreak, a non-migrant man in Accra said that he “had that in mind but I don’t have enough capital to do so. I already had that in mind before the outbreak of the coronavirus”. He told the interviewer that he would be willing to go “anywhere” if he was able. (ACC-NM-M-AA-06) Another non-migrant from Accra shared that she would not take the opportunity to work and live somewhere else due to fears of the virus. The woman said that “[even if [the virus went down] I would, it will be specific country…[a] country with less infection of Covid….I prefer Canada but they are also suffering. The United States of America is my preferred place but things are also not normal there. I don’t think people will like to travel to the USA these days. It will take a long time”. (ACC-NM-F-AA-07).

These observations on abilities to move to highlight the fluid distinctions between the voluntariness of desired immobility—the wish and ability to stay (Carling, 2002; Mata‐Codesal, 2018), the desire but lack of ability to migrate (Carling, 2002), and the lack of aspiration to move combined with an eventual lack of ability to do so (Schewel 2015, 2020).

### Aspirations to move affected by COVID-19 restrictions

Some migrants aspired to continue their migration trajectories, while others reconfigured theirs and returned (temporarily) to previous places of residence. Reasons to move back to the place of origin differed depending on the context and geopolitics of the six cities, but higher-income migrants–whether international or internal–had more abilities to continue their mobility trajectory. The same can be said for lower-income international migrants, who aspired to stay in their places of migration but expressed the wish to be able to visit their family members when COVID restrictions eased somewhat. The results suggest that lower-income internal migrants were more driven by economic needs to aspire to different migration projects.

Respondents from the higher-income international migrant group expressed the desire to stay in their place of residence—the three Europe-based international migrants (AMS-M2-F-RT-03, BRU-M2-M-SND-N2, BRU-M2-M-SND-N5) aspired to exercise their agency to remain in Brussels and Amsterdam:

“I prefer to be where I am at this situation right now. So, I kind of do not want to take any–because I know the situation is still evolving and I’m kind of like–uncomfortable at this juncture. So, I’m not venture out and try to work somewhere else or try something new”. (BRU-M2-M-SND-N5).

For others, the reconfiguration of the migration project included the aspiration to migrate onward. This was particularly the case when migrants perceived that they were not able to meet their broader life aspirations in their place of residence. The Accra-based international importer/exporter wished to resume his business travels from Ghana, but also said: “Living at the right place in the current situation, that’s what I thought, but the current situation has exposed a lot of things; so you feel like you want to move from one place to another place where you can meet your expectation” (ACC-M2-M-AA-01).

Reconfiguring one’s migration project can also mean postponing the decision to migrate, circulate or return (temporarily): “Now, because of the situation, we also were planning to go back to my hometown in India and now we have to postpone it. So that was also something I was looking for and I was a bit upset” (Brussels international migrant BR2).

This can entail waiting until the risks of contracting the virus diminish, economic opportunities arise after the COVID-19 crisis, or travel restrictions are softened. Nearly all of the lower-income international migrant respondents aspired to stay in their migration places while expressing the desire to visit their families in their countries of origin. When asked what he would do if he had an opportunity to work or live elsewhere outside Maputo or outside Mozambique, a Congolese trader responded:

“I like Maputo, I like all of Mozambique. [inaudible] from Mozambique and I liked it. Because nothing very serious has happened to me yet [inaudible] falling into a place and meeting a thug or something, it hasn’t happened to me here in Mozambique yet. This is what I was saying. I like it here in Mozambique because life here in Mozambique is not very complicated either. You can take a cartload for 20 or 10 and eat until the afternoon. Other places, other countries, have places where there are people and you can catch [inaudible] of 20. It is not easy”. (MAP-M2-F-AG-13-DM).

Similarly, higher-income internal migrants preferred to stay where they were, unless they had to travel for work. The Worcester-based CEO of a social service agency scoffed at being asked whether she would like to work and live somewhere else if she had the opportunity:

“It is a stupid question. If I had a choice, I would stay here because I have that choice. So, and I just finished moving and relocating. So, I would stay in Worcester. That doesn’t mean that I wouldn’t pack up and move if an exciting opportunity came across my desk somewhere in the world, I would consider it, but not because I had to or not because I needed to survive”. (WOR-M1-F-JL-07).

Most lower-income internal migrants responded that if they had an economic opportunity elsewhere, they would take it. Reasons included the loss of livelihoods in the city (ACC-M1-F-AA-04), the desire to be closer to family members, sometimes to avoid social isolation (WOR-M1-F-JL-09), and the impossibility of the most deprived to sleep rough during lockdowns: “Some people sleep (…) in the open in the city so when they heard of a possible lockdown they went to their villages and towns where they have a room to sleep” (ACC-M1-M-AA-02). A Dhaka-based hotel cleaner simply said, “If I get working opportunity, I must go.” (DHA-M1-M-MH-02).

The pandemic has prompted the emergence of new factors shaping aspired migration destinations. For instance, in Belgium, rural villages near green areas or in the mountains are preferred to urban centres such as Brussels, but some perceive that the ability to migrate outside the city is a privilege of the rich (BRU-NM-M-SND-N3). Regarding international destinations, some interviewees aspired to migrate to countries where the virus was less widespread or where biosecurity measures met their expectations:

“(…) since we’re in the COVID19 pandemic, I’d have to know how it’s going to be controlled and what kind of security there’s going to be. Now if I know that the sanitation control package is all right, I think there would be no problem. I have no problem going as long as they are complying with the rules, sanitizing, the masks, there would be no problem”. (MAP-M1-M-DM-08).

Nevertheless, looking more closely at the effects of the COVID-19 crisis on future aspirations to migrate, we see that the COVID-19 pandemic does not always affect migration aspirations and decisions. The experience of a migrant born in Cameroon and living in Worcester shows the ambivalence between the aspiration and decision to stay put in his place of residence since the COVID-19 outbreak:

“I think it is quite stressful. It’s quite a burden to think of you being in a part of the world where you can’t just make it back to your family, to your homeland without, you know, connecting to the different services that are interconnected and which are all paralyzed to a certain extent at the moment. Also concerned that going out there [Cameroon], and in an attempt to get there it might also expose you to true risk of contracting the disease, is all part of what preoccupies me right here. (…) I feel like I am safe [in Worcester]. (…) And if the situations get worse in the future what becomes of my family, my kids who are currently living in a region that they know no one, you know, because they had to move out of our region due to the crisis. That is kind of worry to me, you know. So, my safety here, sometimes I feel like maybe it would have been better to be home, to be closer to the kids. To be able to take the right step at any point in time in case of anything. But so far so good. They are fine”. (WOR-M2-M-JL-11).

Non-migrants in the higher-income group expressed different aspirations than low-income non-migrants regarding their desire to stay in their place of origin under the circumstances. When asked about the opportunity to go to work and live somewhere else, either abroad or in their country of origin, our higher-income respondents opted to stay put. A high-income non-migrant man from Accra said, with some hesitation, “Ooohhh oh I’ll prefer to…..I’ll prefer….I’ll….I’ll prefer to stay, I’ll prefer to stay maybe after some while….after some while I’ll travel or so yeah, but for now I’ll prefer to stay.“ (ACC-NM-M-MA-05) Another respondent, a high-income non-migrant woman from Worcester, shared that she judged that:

“there are places that would be you would feel a bit safer like Canada. But, I would like to go to Sweden, I think. But I don’t think there is much safer than we are in terms of Covid. And I don’t really feel like it would be worth the effort of moving because, as you know, I would have a lot of issues, unknown issues, when you move to a new place, and I think it’s better just to stay where you are (WOR-NM-F-JL-04)”.

But lower-income non-migrants expressed a greater desire to move while noting that their capacity was lacking. A non-migrant man from Accra said that he would go “if someone just asked me to come and work for him now… I will go if the person has made payment for my plane ticket…because I would like to change my environment (ACC-NM-M-AA-06). Another low-income non-migrant man from Accra told the interviewer that he would prefer to travel outside the country “because part of my families are there so If I had the opportunity, I would have love to be with them (ACC-NM-M-MA-01)” And a young non-migrant-man-from Brussels, a student, explained that:

“Yeah, of course it was on my plan at first, at the end of my self study to move to Spain, work there, but unfortunately, it’s really difficult to, first, to find a job there. And I just realized it’s really difficult to find a job here too. So right now, because I’m in a, I’m in a sharing flat with my roommates, I want to continue to live with them. I will continue looking for a job here, like for one year. And after that, if I find something and if I have the experience and an opportunity abroad, I will move to Spain (BRU-NM-M-SND-N9).

Aside from the possible constraints to return to the country of origin due to border closures and fears to get infected by the virus, migrants’ ambivalences in migration aspirations are also explained by perceived risks of losing their ability to come back to the host country because of administrative constraints. For some, plans to migrate remained unchanged, while for others the timing of migration and the priorities driving migration did change. The analysis raises questions on which life-course factors lead to stability or change in priorities, aspirations, destinations, and times of reference in the migration decision-making process during COVID-19.

### Differences in mobility decisions across socio-demographic status

The experience of mobility decisions across the wide range of non-migrants, internal migrants, and international migrants reveals differences in impacts across socio-economic status and class across the six cities. General differences in the barriers, ability, and aspirations on mobility decisions across socio-economic status are highlighted in Table 3 for individuals with low and high incomes in the sampled populations of international (foreign-born) migrants and internal migrants, and those non-migrants with long-term residency within the cities.

**Table 3:** Differential impacts of barriers, ability, and aspirations on mobility decisions across socio-economic status in sampled populations.

Socio-economic category	Barriers to movement	Ability to move	Aspirations to move
High-income international migrants (*n* = 4)	Low impact of movement restrictions	Economic ability but restricted choice	Aspirations to stay in the current locality
Low-income international migrants (*n* = 10)	Movement restrictions affecting family life	Perceived stress rather than economic hardship	Aspiration to stay in the current locality
High-income internal migrants (*n* = 8)	Stress, loss of work, police harassment	Fear of virus, restrictions on transportation	Aspiration to stay in the current locality
Low-income internal migrants (*n* = 8)	High-stress levels, an economic situation very challenging	Stress associated with family elsewhere	Would take up economic opportunities elsewhere
High-income non-migrants (*n* = 7)	Privilege and choice to stay or move	Travel restrictions changed the ability to travel for work and holiday	Desire to stay regardless of aspirations
Low-income non-migrants (*n* = 10)	Economic precarity and health	Desired relocation but without capacity	Desire to move but lacking capacity

The core differences in Table 3 are the divergence in the precarity of livelihoods for low-income residents compared to high-income residents, regardless of migration life choice. The data suggest that the emergence of widespread movement restrictions exacerbated already challenging economic situations, although that stress was not limited to economic hardship. Respondents from non-migrant as well as internal migrant groups expressed a wish to move if there were economic opportunities elsewhere, while international migrants wished to continue residing in their current locations.

Overall, high-income respondents across migrant and non-migrant populations did not experience precarity in terms of livelihood. Economic ability was much less affected, although travel restrictions affected leisure choices. In some sites, high-income internal migrants were more impacted by mobility restrictions and loss of clientele. However, all high-income respondents aspired to remain where they were during the pandemic.

In summary, the ability to maintain livelihoods and to plan for the future was curtailed by the pandemic lockdown measures for all respondents both migrants and on-migrants. But the data show that the themes of barriers to movement, abilities to move, and the future aspiration to move are particularly manifest among migrant populations and common across all geographical contexts and levels of social status.

## Discussion and conclusion

The data on the experiences of migrants reported here brings into sharp focus how individual mobility decision-making was affected during the COVID-19 crisis in six cities with diverse migration histories, social and economic development, and across migrants and non-migrants with varying levels of socio-economic status and class.

The results here show that the COVID-19 crisis has reduced the ability and increased the costs of mobility for many. In other words, we show here that the increasing regulations and interventions reduced even further people’s abilities to migrate. These trends also affect people’s aspirations and agency for being and doing what they value in multiple places. The pandemic has significantly altered the balance that many types of migrants, as well as non-migrants, might have reached to deal with contradictory preferences and priorities in their current locations and those of their networks and commitments.

Drawing on the concepts of aspirations and abilities to move (Carling and Schewel, 2018; de Haas, 2021), we have examined three mechanisms through which the COVID-19 crisis may have affected individual mobility decisions: (1) through the direct impacts of barriers to movement (e.g. travel restrictions and border closures), (2) through the impact of the pandemic on abilities to move, and (3) through the impacts of the pandemic on aspirations to move. By acknowledging that aspirations and abilities to move are embedded in macro-level structural, we have explored how structural factors increase the precarity of place (Banki, 2013) for all. These outcomes hamper all residents, whether prior migrants or non-migrants, from improving their circumstances through mobility. The results demonstrate how the three mechanisms interact and are common across social status and locations.

In terms of migration barriers, the respondents highlighted three types of barriers they experienced: (a) direct restrictions due to lockdown policies limiting mobility; (b) fear of contracting the SARS-CoV-2 virus; and (c) obligations to stay put in order to protect family members with poorer health conditions or to comply with expectations of other household members. As such, the COVID-19 pandemic intensified the multiple insecurities inherent to mobility experiences. The pandemic increased perceived and actual constraints to freedom, ranging from physical danger, when people fear contracting the virus and do not feel safe to travel or migrate, to actual mobility barriers due to lockdown policies. Overall, our results highlight that biosecurity considerations and related fears to contract the virus played a large role in mobility decisions during the COVID-19 pandemic. These considerations can be categorized as a subdimension of what Carling (2002) categorises as physical dangers. Every day multi-sited relations between migrants and their family members became disrupted at all income levels, which contributed to migrants’ precarity of place. However, whereas the barriers for higher-income migrants varied across sites and across migrants, lower-income international migrants mainly mentioned the barriers to travel to their countries of origin due to travel bans.

The data reveal significantly altered abilities and aspirations to move among migrant respondents. The results on abilities to move revealed stark contracts between lower and higher-income respondents. The inability of lower-income respondents to move led to involuntary immobility, as it left respondents unable to overcome migration constraints. These constraints mainly referred to the migration barriers described above. Higher-income migrants, as well as non-migrants, most frequently emphasised a lack of agency, resulting from mobility barriers such as lockdown restrictions and threats to health, as the main factor affecting their abilities to move. However, compared to non-migrants in the sample, there appears to be an exacerbation of the vulnerabilities shared by all migrants independently of their profile. Factors such as irregular or temporary administrative status, weaker support networks in the main place of residence, or expectations and obligations to provide support to others transnationally also affected migrants’ freedom to stay in the place of residence or to move.

The study also shows that there is some degree of persistence or even exacerbation in the differences between those with more and those with fewer abilities. Truncated abilities as a consequence of the COVID-19 crisis especially for the most vulnerable are in line with previous work on migration in contexts of environmental disasters, according to which the most vulnerable who need and aspire to migrate tend to lack the ability to do so and become further marginalised.

Finally, aspirations to move and migration projects—involving decisions to stay or move—were often reconfigured because of the perceived mobility barriers and the altered abilities to move. Higher-income migrants displayed higher aspirations to continue their migration projects in the future, whereas lower-income migrants were more likely to alter or postpone their plans to move, driven by economic needs. Reconfigurations of mobility projects took different forms and included onward migration, circulation, return either temporarily or permanently, or postponement of mobility decisions. Most respondents, regardless of their migration or socio-economic status, preferred to stay in their places of current residence, while being able to visit family members abroad if needed, and unless better economic opportunities would arise elsewhere. However, the COVID-19 pandemic also laid bare some new mobility drivers, which included aspirations to move to ‘safer’ countries in terms of health risks, or aspirations to move to perceived greener areas within countries.

The consequences of the COVID-19 pandemic on migrant populations in cities highlight wider lessons for recovery and response. The study here hints at structural changes in the way security, mobility, and migration are perceived. It is only in the long term that we will be able to understand if such changes are temporary or part of deeper social transformations. From an economic perspective, initially, in the pandemic responses, the dominant portrayal of movement as a biosecurity risk gave way to the realisation of how migrant populations, not least in low-paid occupations play a key role in economic functioning.

Furthermore, the dynamism of many city economies is often tied in hidden ways to mobility, aspirations of new populations, and innovation. Economic strategies for pandemic recovery need therefore to address certainty and stability in aspirations to ensure labour and skills availability. Given ongoing uncertainty, this study points to an imperative for integration, both socially and economically, of both migrant and indeed other marginalised social groups to realise goals for safe, sustainable and resilient cities.

## Data Availability

The datasets of interview transcripts generated and analysed during the current study are available from the corresponding author on reasonable request.

## References

[CR1] Adger WN, Safra de Campos R, Siddiqui T (2021). Human security of urban migrant populations affected by length of residence and environmental hazards. J Peace Res.

[CR2] Aiyemo B (2020). Recessions and the vulnerable. World Dev.

[CR4] Asian Development Bank Institute (ADBI), Organisation for Economic Co-operation and Development (OECD) and the International Labour Organization (ILO) (2021) Labour migration in Asia: impacts of the COVID-19 crisis and the post-pandemic future. https://www.adb.org/sites/default/files/publication/690751/adbi-book-labor-migration-asia-impacts-covid-19-crisis-post-pandemic-future.pdf. Accessed 22 Apr 2023

[CR5] Banki S (2013). Precarity of place: a complement to the growing precariat literature. Global Discourse.

[CR6] Boyer F (2005). Le projet migratoire des migrants touaregs de la zone de Bankilaré: la pauvreté désavouée. Stichproben. Wiener Z Krit Afr.

[CR7] Carling J (2002). Migration in the age of involuntary immobility: theoretical reflections and Cape Verdean experiences. J Ethn Migr Stud.

[CR8] Carling J (2014). The role of aspirations in migration.

[CR9] Carling J, Schewel K (2018). Revisiting aspiration and ability in international migration. J Ethn Migr Stud.

[CR10] Carling J, Collins F (2018). Aspiration, desire and drivers of migration. J Ethn Migr Stud.

[CR11] Collyer M, Düvell F, de Haas H (2012). Critical approaches to transit migration.

[CR12] Czaika M, Vothknecht M (2014). Migration and aspirations—are migrants trapped on a hedonic treadmill?. IZA J Migr.

[CR13] Elisabeth M, Maneesh PS, Michael S (2020) Refugees in Sweden during the Covid-19 pandemic—the need for a new perspective on health and integration. Front Public Health 8. 10.3389/fpubh.2020.574334 10.3389/fpubh.2020.574334 PMC7604301 33194974

[CR14] Fereday J, Muir-Cochrane E (2006). Demonstrating rigor using thematic analysis: a hybrid approach of inductive and deductive coding and theme development. Int J Qual Methods.

[CR15] Fernández-Reino M, McNeil R (2020). Migrants’ labour market profile and the health and economic impacts of the COVID-19 pandemic.

[CR16] Gamlen A (2020). Migration and mobility after the 2020 pandemic: the end of an age.

[CR17] Gödecke T, Waibel H (2011). Rural–urban transformation and village economy in emerging market economies during economic crisis: empirical evidence from Thailand. Camb J Reg Econ Soc.

[CR18] González-Leonardo M, López-Gay A, Newsham N (2022). Understanding patterns of internal migration during the COVID-19 pandemic in Spain.. Popul Space Place.

[CR19] Greenaway C, Gushulak B, Bourbeau P (2017). Pandemics, migration and global health security. Handbook on migration and security.

[CR20] IOM (2021) World Migration Report 2022. IOM, Geneva, https://publications.iom.int/books/world-migration-report-2022

[CR21] IOM (2019). World migration report 2020.

[CR22] New York Times (2021). Tracking Coronavirus in Massachusetts: latest map and case count.

[CR23] Greenaway C, Hargreaves S, Barkati S (2020). COVID-19: exposing and addressing health disparities among ethnic minorities and migrants. J Travel Med.

[CR24] Guadagno L (2020) Migrants and the COVID-19 pandemic: an initial analysis. In: Migration research series. International Organization for Migration, Geneva

[CR25] de Haas H (2006). Migration, remittances and regional development in Southern Morocco. Geoforum.

[CR26] de Haas H (2021). A Theory of Migration: the aspirations—capabilities framework. Comp Migr Stud.

[CR27] de Haas H (2003) Migration and development in Southern Morocco. The disparate socio-economic impacts of outmigration on the Todgha Oasis Valley. Ph.D. Dissertation, University of Nijmegen

[CR28] de Haas H (2014) Migration theory: Quo Vadis. International Migration Institute Working Paper 100. University of Oxford

[CR29] Hale T, Webster S, Petherick A (2020). Oxford COVID-19 government response tracker.

[CR30] Hugo G (1996). Environmental concerns and international migration. Int Migr Rev.

[CR31] Içduygu A (2020) Stranded irregular migrant workers during the COVID-19 crisis: the question of repatriation. Research papers series on COVID-19 and its role in the transformation of migration and mobility

[CR32] Lau S, Samari G, Moresky RT (2020). COVID-19 in humanitarian settings and lessons learned from past epidemics. Nat Med.

[CR33] Jolivet (2020). Post‐2008 multi‐sited household practices: between Morocco, Spain and Norway. Int Migr.

[CR34] Lentzos F, Rose N (2009). Governing insecurity: contingency planning, protection, resilience. Econ Soc.

[CR35] Li A, Liu Z, Luo M (2021). Human mobility restrictions and inter-provincial migration during the COVID-19 crisis in China. Chin Sociol Rev.

[CR36] Li J, Rose N (2017). Urban social exclusion and mental health of China’s rural–urban migrants—a review and call for research. Health Place.

[CR37] Lubkemann SC (2008). Involuntary immobility: on a theoretical invisibility in forced migration studies. J Refug Stud.

[CR38] Mata‐Codesal D (2018). Is it simpler to leave or to stay put? Desired immobility in a Mexican village. Popul Space Place.

[CR39] Nimer M, Rottmann SB (2021) Logistification and hyper-precarity at the intersection of migration and pandemic governance: refugees in the Turkish Labour Market. J Refug Stud feab076. 10.1093/jrs/feab076

[CR40] Raju E, Dutta A, Ayeb-Karlsson S (2021). COVID-19 in India: who are we leaving behind?. Prog Disaster Sci.

[CR41] Rao N, Narain N, Chakraborty S (2020). Destinations matter: social policy and migrant workers in the times of COVID. Eur J Dev Res.

[CR42] Richaud L, Amin A (2019). Mental health, subjectivity and the city: an ethnography of migrant stress in Shanghai. Int Health.

[CR43] Rigg J, Salamanca A, Phongsiri M (2018). More farmers, less farming? Understanding the truncated agrarian transition in Thailand. World Dev.

[CR44] Robeyns I (2006). The capability approach in practice. J Political Philos.

[CR46] Sabates-Wheeler R, Feldman R (2011). Migration and social protection: claiming social rights beyond borders.

[CR47] Schapendonk J, van Liempt I, Schwarz I (2020). Re-routing migration geographies: migrants, trajectories and mobility regimes. Geoforum.

[CR48] Schewel K (2015). Understanding the aspiration to stay. A case study of young adults in Senegal.

[CR49] Schewel K (2020). Understanding immobility: moving beyond the mobility bias in migration studies. Int Migr Rev.

[CR50] Schotte S, Danquah M, Osei RD (2021). The Labour Market impact of COVID-19 lockdowns: evidence from Ghana.

[CR51] Sen A (1985). Well-being, agency and freedom: the Dewey lectures 1984. J Philos.

[CR52] Sen A (1999). Commodities and capabilities.

[CR53] Siddiqui T, Szaboova L, Adger WN (2021). Policy opportunities and constraints for addressing urban precarity of migrant populations. Global Policy.

[CR54] Stawarz N, Rosenbaum‐Feldbrügge M, Sander N (2022). The impact of the COVID‐19 pandemic on internal migration in Germany: a descriptive analysis.. Popul Space Place.

[CR55] Suhardiman D, Rigg J, Bandur M (2021). On the coattails of globalization: migration, migrants and COVID-19 in Asia. J Ethn Migr Stud.

[CR56] UN (1998) Recommendations on statistics of international migration. Revision 1. United Nations. Available via https://unstats.un.org/unsd/demographic-social/Standards-and-Methods/files/Principles_and_Recommendations/International-Migration/SeriesM_58rev1-E.pdf. Accessed 25 May 2021

[CR57] UN DESA (2020) International migration 2020 highlights. United Nations Department of Economic and Social Affairs, Population Division. www.un.org.development.desa.pd/files/undesa_pd_2020_international_migration_highlights.pdf. Accessed 25 May 2021

[CR58] UN DESA (2021) World Population Prospects 2021. United Nations Department of Economic and Social Affairs

[CR60] WHO (2021) COVID-19 Intel database. COVID-19 explorer. World Health Organisation

